# Primary Cutaneous Anaplastic Large Cell Lymphoma (pcALCL) in the Elderly and the Importance of Sport Activity Training

**DOI:** 10.3390/ijerph17030839

**Published:** 2020-01-29

**Authors:** Antonello Sica, Paola Vitiello, Andrea Ronchi, Beniamino Casale, Armando Calogero, Evangelista Sagnelli, Gilca Costa Nachtigal, Teresa Troiani, Renato Franco, Giuseppe Argenziano, Elvira Moscarella, Caterina Sagnelli

**Affiliations:** 1 Department of Precision Medicine, University of Campania Luigi Vanvitelli, 80131 Naples, Italy; teresa.troiani@unicampania.it; 2 Dermatology Unit, University of Campania Luigi Vanvitelli, 80131 Naples, Italy; paoladermosun@gmail.com (P.V.); giuseppe.argenziano@unicampania.it (G.A.); elvira.moscarella@unicampania.it (E.M.); 3 Department of Mental Health and Preventive, University of Campania Luigi Vanvitelli, 80131 Naples, Italy; ronchi.andrea@hotmail.it (A.R.); evangelista.sagnelli@unicampania.it (E.S.); renato.franco@unicampania.it (R.F.); sagnelli.caterina@libero.it (C.S.); 4 Department of Pneumology and Tisiology, AO Dei Colli—V. Monaldi, 80130 Naples, Italy; bennycasale@hotmail.com; 5 Department of Advanced Biomedical Sciences, University of Naples Federico II, 80131 Naples, Italy; armando.calogero2@unina.it; 6 Department of Clinical Medicine, Faculty of Medicine at UFPel—Federal University of Pelotas, 1160 Centro, Pelotas, Brazil; gilca.cn@gmail.com

**Keywords:** cutaneous lymphoma in the elderly, skin tumors, T-cell lymphomas, sport activity training

## Abstract

Primary cutaneous anaplastic large cell lymphoma (pcALCL) is part of a spectrum of cutaneous CD30+ lymphoproliferative disease that also includes lymphomatoid papulosis. It often occurs in elderly patients, presenting at a median age of 60 years, although it may occur at any age. It is a CD30+ T-cell neoplasm composed of large cells with anaplastic, pleomorphic, or immunoblastic morphology, with exclusively cutaneous onset and localization. The clinical course of pcALCL is predominantly indolent. Most elderly patients with lymphoma tend to have a sedentary lifestyle, which has a negative effect on their quality of life (QoL) and survival. Several studies indicate that exercise has a positive impact on QoL because it reduces peak oxygen consumption, improves physical capacity, increases self-esteem, reduces accumulated stress, and promotes relaxation. Therefore, particularly in indolent lymphomas, it is necessary to indicate a program of physical activity to be practiced systematically. Complete surgical excision and local radiotherapy are the first line gold standard in pcALCL with a solitary lesion.

## 1. Introduction

Primary cutaneous anaplastic large cell lymphoma (pcALCL) is a CD30+ T-cell neoplasm composed of large cells with anaplastic, pleomorphic, or immunoblastic morphology, with exclusively cutaneous onset and localization [1]. The clinical course of pcALCL is predominantly indolent, completely different from that of the systemic forms of anaplastic large cell lymphomas (ALCL) [2]. ALCL are a group of T-cell lymphoproliferative diseases characterized by the presence of anaplastic cells with CD30 positivity and a variable expression of T-cell markers [3]. Neoplastic cells defined as “hallmark cells” are morphologically distinguishable as large pleomorphic cells with abundant cytoplasm and eccentric kidney-shaped nuclei. These are common aspects of all kinds of ALCL, but each type differs in clinical presentation, prognosis, and molecular features. According to the most recent revision of the WHO (2016) [4], ALCL are classified as anaplastic lymphoma kinase ALK-positive large cell anaplastic lymphoma (ALK+ ALCL), ALK-negative ALCL (ALK- ALCL), breast-implant-associated ALCL (BI-ALCL), or pcALCL. The first two forms have a systemic clinical presentation and development with lymphadenomegaly, splenomegaly, hepatomegaly, and secondary extranodal infiltration mainly involving the skin, bones, soft tissues, and lungs, and are associated with systemic symptoms such as fever, weight loss, and night sweats. Central nervous system involvement is rare. At the onset of illness, it is common to find an already widespread disease in stage III–IV with systemic symptoms, according to the Ann Arbor staging system. ALK+ ALCL is more common in adolescents and children. ALK- ALCL has a higher incidence in adults over 60 years. Both forms are aggressive, with ALK+ ALCL in young people being responsive to chemotherapy and showing an approximate 70% long-term survival, while ALK- ALCL, usually seen in older adults, has a less favorable prognosis. Systemic ALK- ALCL with rearrangement in the DUSP22-IRF4 locus have a more favorable prognosis, while those with TP53 rearrangements have a poor prognosis.

BI-ALCL is a form localized in areas adjacent to the breast implant. It generally has an indolent course and a good prognosis. Morphologically comparable to the other forms, it is ALK- with an excellent response to surgical therapy. However, systemic development of this type is also known to occur, characterized by an unfavorable prognosis similarly to the above-described systemic forms.

Patients with pcALCL are frequently diagnosed at an older age, but it may also present in young people. Males are more often affected than females (a ratio of 3:1).

About 25% of patients have the DUSP22-IR4 locus at onset, while TP63 rearrangements are rare. Unlike systemic forms, these chromosomal aberrations do not appear to be related to a worse prognosis [5].

Support in patients with lymphoma is essential to avoid depression, reduction of self-esteem, and the onset of unreal emphasis of symptoms such as fatigue and pain [6,7]. It is striking how most cancer patients have a sedentary lifestyle, which can have a negative effect on their quality of life (QoL) [8,9]. In addition, several studies indicate that physical activity has a positive impact on QoL in cancer survivors [10,11], because it reduces peak oxygen consumption, improves physical capacity, increases self-esteem, reduces accumulated stress, and promotes relaxation [12]. Physical activity also exerts a favorable effect on metabolism, inflammation, and the immune system [13]. In particular, it regulates macrophages and the natural killer lymphocytes that are widely involved in interactions with cancer cells [14,15,16]. In addition, randomized studies have highlighted how physical activity in cancer patients may improve QoL [17,18,19]. In our department, we encourage patients to progressively increase and take part in a program of physical activity depending on their physical abilities and attitudes. Such physical activity includes sports activities, planned exercise, domestic activities, professional activities, and walks. The main objective of this review will be to describe all new diagnostic and therapeutic features of primary cutaneous ALCL regarding elderly patients and to evaluate the importance of physical activity.

## 2. Clinical Presentation

**Figure 1:**
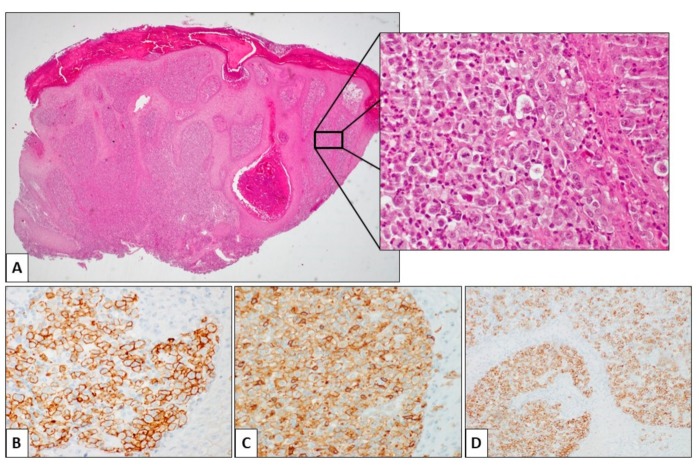
Histological features of primary cutaneous anaplastic large cell lymphoma (pcALCL). (**A**) This skin punch biopsy is characterized by a dense lymphoid population filling the dermis, without significant epidermotropism. The epidermis shows secondary changes, including hyperkeratosis and papillomatosis (Hematoxylin and eosin (H&E), 2.4×). Inset: the neoplastic population is composed of large-sized cells with abundant, slightly eosinophilic cytoplasm and roundish, atypical nuclei. Two mitotic figures are evident in the center of the field. Some neutrophils and eosinophils are scattered in the context of the neoplastic population (H&E, 20×). The large cells are positive for CD3 (**B**), CD4 (**C**), and CD30 (**D**) immunohistochemical staining.

**Figure 2:**
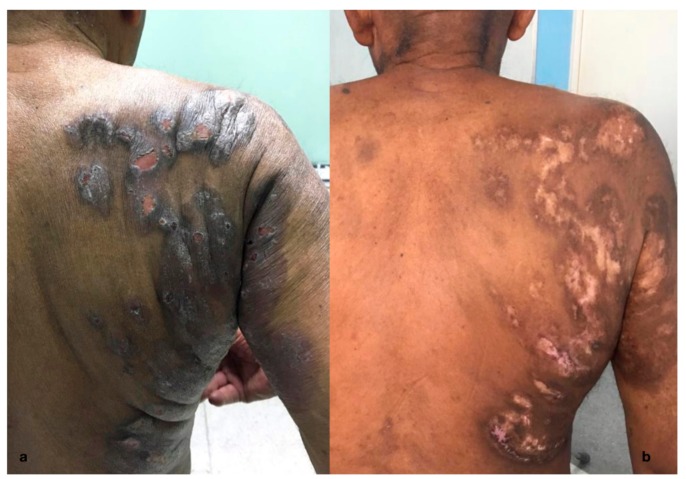
Clinical presentation of ALK- pcALCL with extensive ulcerated plaques located on the trunk and upper arms (**a**). Resolution of skin lesions, with central scarring and depigmentation after treatment (**b**).

For most patients, the disease begins with a single skin nodular lesion (Figure 1), often ulcerated, with a diameter of more than 2 cm, while for 20% of patients, the onset is multiple localizations on different skin areas (Figure 2). The clinical features of skin lesions are nodules, papules, plaques, and cellulitis-like ulcerations. Their color ranges from red to violaceous and may rapidly grow and have predilection for the head, neck, and extremities. Generally, the disease is asymptomatic and fevers, night sweats, or weight loss are usually lacking. Spontaneous regressions have been reported for 10%–42% of lesions. PcALCL may be the result of mycosis fungoides (MF) progression [20]. Therefore, in patients with pcALCL, a current or previous diagnosis of MF should be excluded. The differential diagnosis between pcALCL and CD30+ MF transformed can be even more difficult when there is a clinical presentation with a previous or simultaneous patch–plaque MF stage. The presence of multiple lesions in 20% of the cases can make the differential diagnosis difficult with the type C Lymphomatoid papulosis (LyP), which presents borderline lesions [20,21]. The onset with multiple sites and major extension appears to have a worse prognosis due to a higher incidence of cases with evolution to secondary cutaneous ALCL (scALCL). The International Society of the Cutaneous Lymphomas European Organization for Research and Treatment of Cancer proposed the tumor, node, metastases (TNM) classification of cutaneous lymphoma other than mycosis fungoides and Sezary syndrome (Table 1) [20]. This classification is based on the size of the lesions, the skin areas involved, the lymph node stations involved, and the extranodal and visceral localizations. It is clearly usable for the staging of both pcALCL and scALCL. Staging is essential to distinguish the pcALCL from scALCL (Table 1). All patients at diagnosis should perform a complete metabolic panel, lactate dehydrogenase (LDH), and Tc-PET, since these exams allow for the exclusion of a systemic infiltration from ALCL.

### 2.1. Histological and Molecular Features

**Figure 3:**
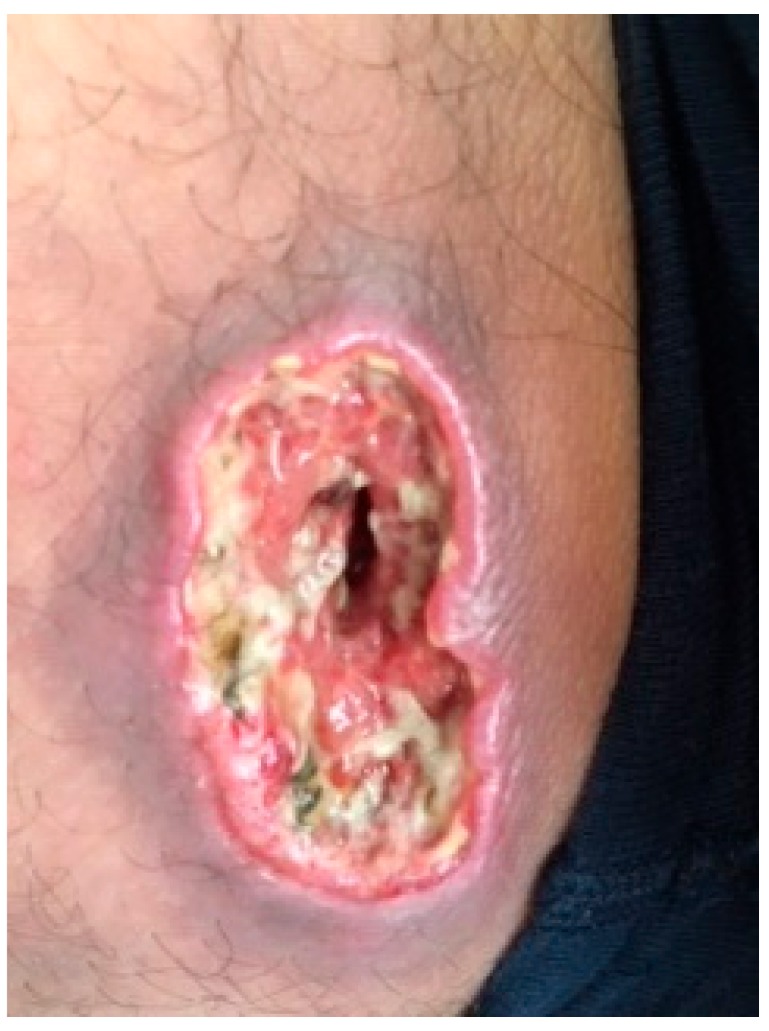
ALK- pcALCL single localization on a leg.

The classical form of pcALCL is characterized by a nodular infiltrate of large lymphoid cells, mostly confined to the dermis, and usually without epidermotropism (Figure 3). However, several histological variants have been described as rich in neutrophils and eosinophils (scattered in the classical form), which are more common in immunosuppressed patients: the angiocentric form, the angiodestructive form, the subcutaneous and keratoacanthoma-like form, the sarcomatoid variants with prevalent spindle-cell morphology, the small cells, and the intravascular ALCL form. The cell phenotype is usually characterized by a positivity for CD30 (75% in tumor cells), CD3, CD4, CD45RO, and no expression for CD5 and CD2. In about 50% of cases, it has been described as positivity for CD71, HLA-DR and CD25 (IL-2R), TIA1, perforin, and granzyme B for the cutaneous lymphocyte antigen (CLA, HECA-452). Positive Epithelial Membrane Antigen (EMA) is only focal, unlike the systemic ALK- ALCL, which strongly expresses this marker. KIR3DL2 (CD158k) from neoplastic cells is expressed as in Sezary syndrome. Another important feature that is very useful for diagnostics is the alteration of the receptor complex of T/CD3 cells and the transcription factors associated with T receptors and signal transduction molecules—a frequent finding in CD30+, systemic, and cutaneous lymphoproliferative diseases. The monoclonal rearrangement of the TCR gene is present in 65%–90% of cases. The majority of pcALCLs are ALK-, DUSP22-, and TP63-. However, other mutations may also occur, particularly considering the frequency order: DUSP22 rearrangement, ALK translocations, TP63 rearrangements, and NPM1–TYK2 gene fusion [22,23]. The DUSP22 rearrangements are related to a particular histological pattern characterized by the simultaneous presence of two different clones: one large CD30+ cell infiltrating the dermis, and another characterized by small CD30+ lymphoid cells with a pattern of pagetoid reticulosis. It has been shown that the expression of the chemokine receptor gene CCR8 is associated with DUSP22 rearrangements in ALCL. It is possible that the higher expression level of this receptor will explain the lower tendency of pcALCL to disseminate in extracutaneous sites contained by the limiting action of the immune system. The presence or absence of the DUSP22 rearrangement does not change the prognosis. In contrast to systemic anaplastic large-cell lymphoma (sALCL), the cases of ALK+ pcALCL seem to have a favorable outcome, comparable to that of patients with ALK- pcALCL. The TP63 rearrangement has long been studied because it has previously been associated with very aggressive cases of the disease. Subsequent studies do not seem to have confirmed the specificity of this rearrangement in the rapidly evolving forms. Other mutations seem to have a relationship with the evolution of the disease, including those on the path JAK1/STAT3. These mutations were found in only 5% of pcALCL. It is well known that deregulation of Notch signaling in hematopoietic cells is linked to the development of various hematological malignancies, including chronic B-cell lymphocytic leukemia, acute T-cell lymphoblastic leukemia, acute myeloid leukemia, multiple myeloma, sALCL, and Hodgkin’s lymphoma. In addition, the pcALCL neoplastic cells have an increased expression of the intracellular domains of Notch1, Notch2, Notch3, and Notch4 receptors, as well as of the HES1 product and the Delta ligand. The inhibition of gamma-secretase with specific inhibitors has been shown to induce apoptosis and decrease cell viability in the pcALCL cell lines, inhibiting the Notch pathway. Therefore, this is a potential target to be considered in the near future for the therapies of these diseases.

### 2.2. Cytogenetic Alterations

The following chromosomal alterations are characteristic of and recurrent in this type of lymphoma: the gains of 7q31 and losses in the 6q16–6q21, 6q27, and 13q34 regions.

## 3. Differential Diagnosis

The most important differential diagnosis for a pcALCL is with the sALCL. Indeed, what appears to be an apparently isolated skin involvement may also be a skin localization of systemic disease. Consequently, the first step for a definitive diagnosis of pcALCL is the exclusion of sALCL. The morphology and immunophenotypic characteristics of LyP (in particular type C) and pcALCL overlap significantly and no biomarker has to date been able to reliably distinguish these two entities, so it is essential to correlate the pathological results with the clinical history that represents the only distinctive elements. In fact, it is the clinical behavior of LyP, characterized by recurrent episodes and the onset of papules and nodules sometimes at spontaneous resolution, which helps in the distinction between the two pathologies. Another very similar pathology for clinical presentation is MF with large cell transformation (MF-LCT), among other things, such as CD30+. From a histopathological point of view, MF-LCT is characterized by epidermotropic or dermal aggregates of CD30+ tumor cells that occur less frequently in pcALCL. The expression of GATA3+ is more frequent in MF-LCT, while the perforin is more expressed in pcALCL. Adult T-cell lymphoma (ATCL) may show diffuse CD30 expression and nuclear pleomorphism similar to pcALCL, but the presence of HTLV-1 in the former is pathognomonic. Other entities that enable the differential diagnosis of pcALCL include B-cell lymphoma and cutaneous leukemia. Some cases of diffuse large cell B-lymphoma CD30+ of an anaplastic variant may represent a diagnostic challenge, especially if CD20 and CD79a are negative. Classical syncytial nodular sclerosis Hodgkin’s lymphoma (NSCHL) is another disease to consider, although pcALCL can rarely express CD30 and CD15 simultaneously, and CD45 is positive in most pcALCL and negative in NSCHL. Leukemia cutis may also show a pcALCL-like histology but usually expresses TdT, CD34, and/or CD117, and myeloid lineage markers such as myeloperoxidase.

## 4. Therapies

Complete surgical excision and local radiotherapy are the first line gold standard therapies of pcALCL with solitary or grouped localized lesions (up to T2N0M0) [24]. In the more advanced stages of the illness, with cutaneous dissemination and in the relapsing/refractory disease, the administration of brentuximab vedotin (BV), an anti-CD30 antibody-conjugate, has shown low toxicity and a high percentage of remissions and has been recommendable in the elderly patient [25,26]. Methotrexate and bexarotene are further indicated therapeutic options [27,28,29]. Appropriate screening for Hepatitis C virus (HCV) and Hepatitis B virus (HBV) infection should be performed to prevent any flare-ups of viral hepatopathies [30,31,32,33,34]. The γ-secretase inhibitors (inhibition of the Notch pathway) and the JAK1/2/3 inhibitors that are effective in vitro to control the cell growth of the pcALCL have also been suggested for the more advanced stages of the illness [29,34]. Other proposed treatments are the anti-ALK molecules, such as crizotinib, alectinib, and ceritinib, which in pcALCL patients with ALK rearrangements could downregulate the STAT3 pathway, inducing apoptosis. The IPH4102, a humanized monoclonal antibody directed against the KIR3DL2 cell receptor (CD158K), has also been proposed. Finally, histone deacetylase (HDAC) inhibitors (romidepsin and vorinostat) and demethylating agents have demonstrated efficacy in inducing cell cycle arrest, differentiation, and/or apoptosis of tumor cells [35,36,37,38].

## 5. Discussion 

PcALCL is a lymphoma with a good prognosis and low mortality, which mainly concerns the elderly. Unfortunately, several patients have had multiple relapses throughout their clinical history, particularly in forms with diffuse skin localization in multiple body districts [39]. Many therapeutic lines, therefore, characterize the history of these patients. The chemotherapy protocols used for this subset of patients are variable. Cyclophosphamide, doxorubicin, vincristine, and prednisone (CHOP) is one of the most widely used protocols and has an overall response rate (ORR) of 85%. Other regimens well-tolerated by the elderly with comorbidities are cyclophosphamide, vincristine, and prednisone (CVP) oral etoposide, gemcitabine, and methotrexate in low doses or in monotherapy [40,41]. All these regimens are valid therapeutic options in this subset of patients, although they have shown a high frequency of relapses. The efficacy of BV that has a 75% ORR seems different, with a fast response and good tolerability even in older patients. The most common adverse effect described is peripheral sensory neuropathy. The diagnosis of pcALCL is particularly difficult because its clinical presentation is variable. In addition, its histological and molecular characteristics show numerous affinities with similar lymphoproliferative pathologies where the prognosis and therapies are completely different. For this reason, it is important to study the patient from an anamnestic and clinical point of view to arrive at a correct diagnosis assisted by all available molecular, genetic, and histological investigations. The therapy should also consider eventual comorbidities and physical activity. 

## 6. Conclusions

A physical activity program should be recommended to elderly patients with the aim to generate positive feelings and optimism and consider the disease as a moment of transition to face and overcome. It is not easy for those patients who already suffer from comorbidities [39,40,41,42], but it helps to keep the musculoskeletal and circulatory system in good functional condition, to eliminate excess body fat, and to stimulate the regulatory function of the immune system on macrophages, NK cells, cytokine production, and other factors involved in cancer prevention. 

In addition, movement improves energy and hormone metabolism, reduces inflammation, and, finally, helps the patient to stay in good shape with improved self-esteem [42]. Most failures due to discontinuation of chemotherapy or reduction of the standard dosage are generally caused by clinical complications in patients undergoing treatment or by reduced performance status. Improved QoL and cardiovascular status predispose the patient to fully cope with the necessary chemotherapy protocol and, consequently, the likelihood of a successful outcome is increased [43,44,45,46,47,48]. There is no clear evidence of a direct relationship between physical activity and remission in pcALCL, but it is certainly an excellent support for specific treatments of this oncological pathology and all other indolent neoplasms in elderly patients [43,44,45,46,47,48].

The improvement in the general condition of the patients has a significant impact on the therapeutic program and, consequently, can favorably affect the prognosis.

## Figures and Tables

**Table 1:** International Society for Cutaneous Lymphomas (ISCL)/European Organization of Research and Treatment of Cancer (EORTC) tumor, node, metastases (TNM) classification of cutaneous lymphomas other than mycosis fungoides (MF)/Sézary syndrome (SS).

Skin (T)	T1		Solitary Skin Involvement
		**T1a**	Solitary lesion <5 cm in diameter
		**T1b**	Solitary lesion ≥5 cm in diameter
	**T2**		Regional skin involvement: multiple lesions limited to one body region or two contiguous body regions
		**T2a**	All disease encompassing a <15 cm diameter circular area
		**T2b**	All disease encompassing a 15 to ≤30 cm diameter circular area
		**T2c**	All disease encompassing a ≥30 cm diameter circular area
	**T3**		Generalized skin involvement
		**T3a**	Multiple lesions involving two noncontiguous body regions
		**T3b**	Multiple lesions involving ≥3 body regions
**Lymph nodes (N)**	**N0**		No clinical or pathologic lymph node involvement
	**N1**		Involvement of one peripheral or central lymph node region that drains in an area of current or prior skin involvement
	**N2**		Involvement of ≥2 peripheral or central lymph node regions or involvement of any lymph node region that does not drain in an area of current or prior skin involvement
	**N3**		Involvement of central lymph nodes
**Viscera (M)**	**M0**		No evidence of extracutaneous non-lymph node disease
	**M1**		Extracutaneous non-lymph node disease present

Adapted from Kim YH, Willemze R, Pimpinelli N, et al. TNM classification system for primary cutaneous lymphomas other than mycosis fungoides and Sézary syndrome: a proposal of the International Society for Cutaneous Lymphomas (ISCL) and the Cutaneous Lymphoma Task Force of the European Organization of Research and Treatment of Cancer (EORTC). Blood 2007;110(2):480. [21].
